# Optimal echo time for functional MRI of the infant brain identified in response to noxious stimulation

**DOI:** 10.1002/mrm.26455

**Published:** 2016-09-21

**Authors:** Sezgi Goksan, Caroline Hartley, Samuel A. Hurley, Anderson M. Winkler, Eugene P. Duff, Mark Jenkinson, Richard Rogers, Stuart Clare, Rebeccah Slater

**Affiliations:** ^1^ Department of Paediatrics University of Oxford Oxford United Kingdom; ^2^ Nuffield Department of Clinical Neurosciences University of Oxford Oxford United Kingdom; ^3^ Nuffield Department of Anaesthetics University of Oxford Oxford United Kingdom

**Keywords:** BOLD, brain, echo time, imaging, *T*${\;}_{2}^{*}$, infants, pain

## Abstract

**Purpose:**

Blood oxygen level dependent (BOLD) brain activity, measured using functional MRI (fMRI), is dependent on the echo time (TE) and the reversible spin–spin relaxation time constant ( $T_{2}^{*}$) that describes the decay of transverse magnetization. Use of the optimal TE during fMRI experiments allows maximal sensitivity to BOLD to be achieved. Reports that $T_{2}^{*}$ values are longer in infants (due to higher water concentrations and lower lipid content) have led to the use of longer TEs during infant fMRI experiments; however, the optimal TE has not been established.

**Methods:**

In this study, acute experimental mildly noxious stimuli were applied to the heel in 12 term infants (mean gestational age = 40 weeks, mean postnatal age = 3 days); and the percentage change in BOLD activity was calculated across a range of TEs, from 30 to 70 ms, at 3 Tesla. In addition, $T_{2}^{*}$ maps of the whole brain were collected in seven infants.

**Results:**

The maximal change in BOLD occurred at a TE of 52 ms, and the average $T_{2}^{*}$ across the whole brain was 99 ms.

**Conclusion:**

A TE of approximately 50 ms is recommended for use in 3T fMRI investigations in term infants. Magn Reson Med 78:625–631, 2017. © 2016 The Authors Magnetic Resonance in Medicine published by Wiley Periodicals, Inc. on behalf of International Society for Magnetic Resonance in Medicine.

## INTRODUCTION

The blood oxygen level dependent (BOLD) signal detected by functional MRI (fMRI) is dependent on the echo time (TE) used for acquisition, which is in turn related to the $T_{2}^{*}$, a time constant that describes the decay of transverse signal in the presence of magnetic field inhomogeneity and susceptibility effects. Theoretically, maximum BOLD sensitivity will be recorded when the TE is approximately equal to $T_{2}^{*}$ 1, 2, 3. In practice, physiological and thermal noise mean that this relationship is often not directly maintained 4; instead, the optimal TE is the time at which the maximal change in BOLD signal is observed. Although various studies have reported average $T_{2}^{*}$ values in adult gray and white matter ranging from 41 to 66 ms 4, 5, 6, a TE of 30 ms is most often used in adult fMRI studies at 3 Tesla (T) across a range of stimulus modalities and during resting state 7, 8.

Optimization of the TE for use in infants is important given the differences between the adult and infant brain. As the infant brain has higher water content and lower lipid concentrations due to immature myelination and synaptic formations compared with adults 9, 10, the $T_{2}^{*}$ (and therefore TE) should theoretically be longer. Experimentally, $T_{2}^{*}$ values have been shown to be longer in the infant brain compared with adults: at 1.5T, $T_{2}^{*}$ values were reported as being two times longer in both gray and white matter regions compared with adults 11; and at 3T, $T_{2}^{*}$ values of between 102 and 120 ms have been reported in the motor cortex in a single infant 12, which is again approximately double those reported in adults 4, 5, 6. These observations have led to recommendations that a longer TE should be used in infant fMRI 11. Although TEs of 40 and 45 ms have been used for infant fMRI studies conducted at 3T 13, 14, 15, no rigorous assessment of the optimum TE for this group has been conducted.

Despite the challenges of acquiring fMRI data in the infant population, these techniques provide a tremendous opportunity to investigate the brain activity associated with sensory stimulation in the newborn infant. A recent fMRI study investigating the pattern of brain activity that was evoked following noxious stimulation demonstrated that brain regions thought to encode the sensory and affective components of pain, such as the anterior cingulate, insula, and somatosensory cortices, are active in infants 15. In adults, noxious‐evoked changes in brain activity correlate with subjective reports of pain 16 and are altered by analgesics 17, 18, 19, 20. Furthermore, fMRI‐based measures that predict pain intensity in individual subjects have been developed 20 and proposed as a key method for identification of new analgesics 21. The possibility of developing these measures in the infant population is of particular interest because infants cannot describe their pain experience; their pain management and treatment is entirely dependent on surrogate measures of pain 22. However, in order to develop these measures, it is necessary to optimize scanning parameters for the infant population.

In this study, a series of fMRI experiments was performed during which mildly noxious acute experimental stimulation was applied to the sole of the infant's foot while data were acquired across a range of TEs, from 30 to 70 ms. The aim was to establish the TE at which the maximum percentage change in BOLD was observed, across the whole brain and in specific anatomical brain regions. In addition, $T_{2}^{*}$ values were calculated, allowing the relationship between $T_{2}^{*}$ and TE to be investigated in this population at 3T.

## METHODS

### Participants

Fourteen infants, born at term age (37–42 weeks gestational age) were recruited from the Maternity Unit at the John Radcliffe Hospital, Oxford, United Kingdom. All infants were studied within the first 5 postnatal days. Ethical approval (National Research Ethics Service, REC reference: 12/SC/0447) and informed written parental consent were obtained prior to each study. The study was carried out in accordance with the standards set by the Declaration of Helsinki and Good Clinical Practice guidelines.

Infants were eligible for inclusion in the study if they were self‐ventilating in air and clinically stable. Infants were excluded if there were any indications of neurological abnormality or any contraindications for MRI. To reduce the scanning time and exposure to the experimental stimulation, the number of functional echo planar imaging (EPI) sequences was limited such that a maximum of three EPI sequences for the investigation of optimal TE were reported per infant (Table 1). Following data acquisition, one infant was excluded from further analysis due to excessive movement artifacts observed in the EPI. We therefore only consider the remaining 13 infants hereafter. A subset of the data (n = 3) formed part of a dataset previously reported by Goksan et al. in 2015, where brain regions activated in response to noxious stimulation in infants were identified 15.

**Table 1:** Infant Demographics and Information on the Functional Images Acquired per Infant for Each Study

Demographics				Functional Scans					
Infant	Gender	GA at Study (weeks + days)	PNA at Study (days)	Study 1: TE Investigation (ms)					Study 2: $T_{2}^{*}$ Relaxometry
				30	40	50	60	70	
01	M	37 + 4	3	✓^3^	✓^2^	✓^1^			
02	F	37 + 5	4	✓^3^	✓^2^	✓^1^			
03	M	38 + 4	2				✓		✓
04	M	39 + 1	5				✓		✓
05	F	39 + 3	2	✓^3^		✓^2^		✓^1^	
06	M	39 + 5	1				✓		
07	M	39 + 6	2		✓^1*^	✓^2^			✓
08	M	40 + 2	5		✓^1*^			✓^2^	
09	M	40 + 5	4			✓			✓
10	M	41 + 5	4	✓^3^	✓^2^			✓^1^	
11	M	41 + 5	3		✓^1*^	✓^2^			✓
12	F	41 + 5	5						✓
13	F	42 + 5	2				✓		✓

The order in which the echo planar imaging sequences were acquired in study 1 is indicated by the superscript. (*) Indicates data reported in Goksan et al. [15].

F, female; GA, gestational age; M, male; PNA = postnatal age; TE, echo time.

### Study Protocol

The recruitment and MRI study preparation methods used for this investigation have been described in Goksan et al. in 2015 15.

#### Study 1: Investigating Optimal Echo Time

Echo planar imaging data were collected across a range of TEs (30, 40, 50, 60, and 70 ms) in order to investigate the optimal TE. A maximum number of three EPI scans, each with a different TE between 30 and 70 ms, was collected per infant (Table 1). Overall, 12 infants had 23 functional scans that were included in the analysis for Study 1 (see Table 1). Each EPI scan lasted approximately 6 minutes, during which time a mildly noxious acute experimental stimulus (PinPrick Stimulators, MRC Systems, Heidelberg, Germany), calibrated to a force of 128 mN, was applied to the heel of the infant's left foot. Ten stimuli were applied per individual EPI acquisition, with a minimum interstimulus interval of 25 seconds. The interval was chosen based on the neonatal‐specific hemodynamic response function (HRF) described by Arichi et al. 23; the time was extended if necessary to ensure that the infant was settled at the time of stimulation.

#### Study 2: Quantitative $T_{2}^{*}$ Relaxometry

Eight infants were scanned as part of Study 2. One infant was then excluded from this analysis due to excessive motion artefact, leaving seven infants in study 2 (see Table 1). Gradient echo EPI data of the whole brain were acquired across a range of TEs (30, 40, 50, 60, 70, and 80 ms). Two consecutive volumes were collected at each TE so that 12 volumes were collected in total. Six separate sequences were acquired, and each independent sequence consisted of two volumes. All data were acquired at rest and at the start of the scanning session (either immediately before or after a structural scan).

### MR Image Acquisition

MR data were acquired using a Siemens 3T Magnetom Verio system (Erlangen, Germany) with a 32‐channel adult head coil. Structural T_2_‐weighted turbo‐spin‐echo scans were acquired with the following sequence parameters: pulse repetition time (TR) = 13,871 ms; TE = 89 ms; flip angle 150°; resolution 1 mm^3^; slices = 80, and echo train length = 8. If excessive motion was identified, a second structural scan was attempted and the best image was chosen for further analysis. All functional images were acquired using $T_{2}^{*}$‐weighted EPI sequences. Shimming was carried out using the vendor in‐built protocol, and dummy scans were collected at the start of each sequence. Each EPI sequence was collected using various TEs according to the study protocol (Study 1: TE range: 30–70 ms [see Table 1]; TR range: 2,170–3,490 ms; average number of volumes = 124 [range: 96–157]. Study 2: TE range: 30–80 ms; TR = 3,800 ms; 12 consecutive volumes collected, two at each TE). All EPI sequences also had the following parameters: flip angle = 90°, field of view = 192 mm, imaging matrix 64 × 64, resolution 3 mm^3^, number of slices = 33. Prospective acquisition correction for head motion (PACE) was applied during all EPI scans in study 1. PACE is a motion‐correction technique that tracks the position of the head during scan acquisition and applies a real‐time correction for large head movements 24.

For study 1, all EPI data were collected using the shortest TR at the corresponding TE. Whilst the differences in TR may result in a bias toward shorter TEs, the shortest TR was chosen because, when collecting fMRI data in infants, obtaining the shortest possible scanning time is important in order to minimize the chances of excessive motion artifacts. The results from using the shortest TR therefore equate to results that can be achieved from the best overall scanning parameters. In addition, a constant flip angle was used across all EPI sequences despite variation in the TR. This may have resulted in a small loss in the signal‐to‐noise ratio but is unlikely to dramatically alter the findings.

### Data Analysis

#### Study 1: Investigating Optimal Echo Time

All functional MR data processing was conducted using Functional MRI of the Brain's Software Library (FSL) (www.fmrib.ox.ac.uk/fsl) 25, 26, 27. All first‐level analyses were conducted as described in Goksan et al. in 2015, including data preprocessing, methods to minimize motion artefact, and registrations 15. Registration steps included registering an infant's functional image to the structural image and then to two template images; the first corresponding to the infant's gestation in weeks at the time of the scan and finally to a 40‐week gestation common template 28. The fMRI Expert Analysis Tool (FEAT) was used to run statistical modeling on the fMRI data. Time‐series analyses were performed using a general linear model (GLM) by convolving the timing of each noxious stimulus with a neonatal‐specific HRF (gamma function, mean = 7 s; standard deviation [SD] = 3.5 s; described by 23). The noxious stimuli were time‐locked to the fMRI recording using Presentation software (Neurobehavioral Systems Inc., Berkeley, CA). The time was recorded when the experimenter pressed a button concurrent with the time that the experimental stimulus was applied to the participant's foot.

The mean percentage change in BOLD was reported per individual acquisition and was calculated across the whole brain or in a specific region of interest (ROI), detailed below. Featquery was used to obtain a mean percentage change in BOLD following the noxious stimuli, relative to the global mean. Subsequently, a second‐order polynomial was fitted to the individual data points. This was based on previous research describing the relationship between TE and the BOLD response 3, 4, 29. The fitted curve was used to identify the optimal TE, defined as the TE where the maximal change in BOLD response occurred. In addition, the relationship between the contrast‐to‐noise ratio (CNR) and the TE was investigated to ascertain whether these results were consistent with those obtained when considering the maximal change in BOLD response. Parameter estimates were obtained from the GLM, which described the maximal change in BOLD following noxious stimulation within each voxel. This was divided by the SD of the residuals to calculate the CNR across the whole brain.

Where the mean percentage change in BOLD was calculated across the whole brain, a mask excluding voxels of cerebrospinal fluid (CSF) was used. The whole brain and CSF masks were available as part of the neonatal‐specific template corresponding to a 40‐week gestation infant 28. The mask of the CSF regions was subtracted from the mask of the whole brain, and the resulting image was transformed from the neonatal‐specific template onto each individual subject's functional image using inverted transformation matrices generated by the registration steps described above. Following the transformation, the outer edge of the mask was eroded by a single voxel using fslmaths to ensure that all voxels outside of the brain were excluded because these could bias the optimal TE calculation. Finally, fslmaths was used to remove all remaining partial volume voxels between template and native functional space before the resulting mask was input into Featquery.

**Figure 1:**
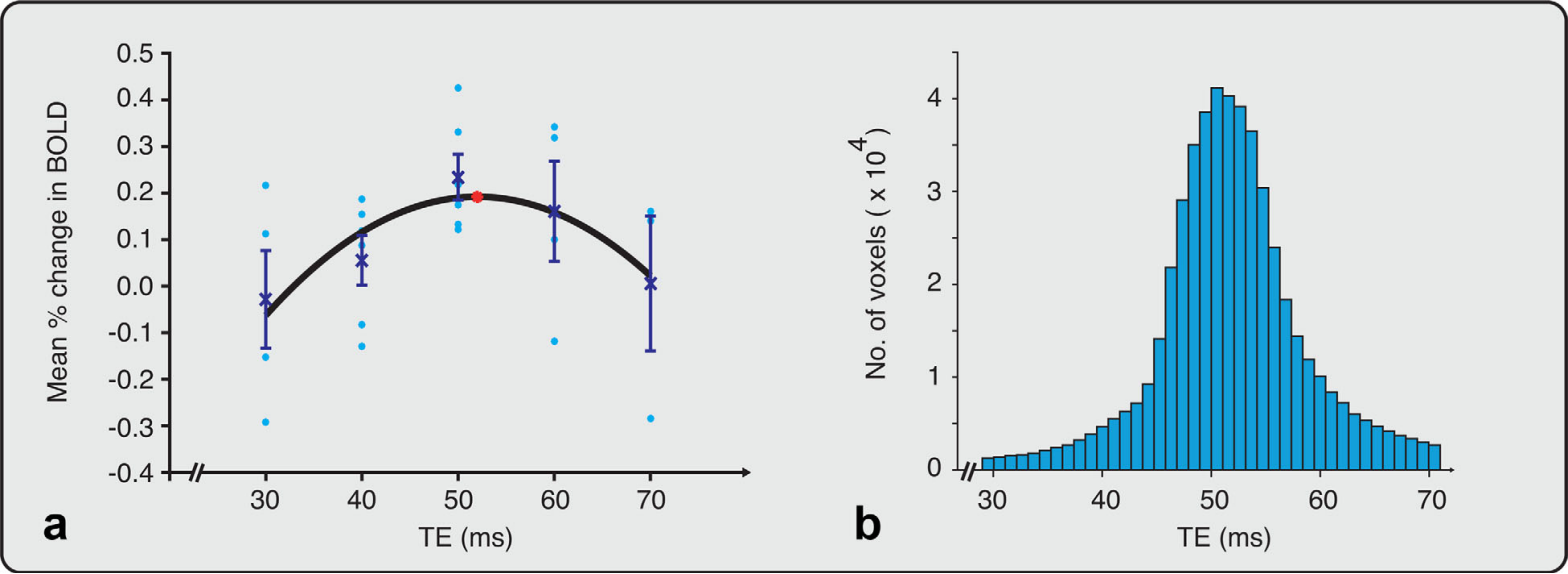
Calculation of the optimal TE across the whole brain. (a) Mean percentage change in BOLD was calculated from EPI data collected at five different echo times (n = 23, TE range: 30–70 ms). The mean (crosses) and standard error of the mean are indicated at each TE. A second‐order polynomial was fitted to the data (black line). An asterisk (red) identifies the TE at which the fitted curve is maximal (TE = 52 ms). The circles (light blue) represent the mean percentage change in BOLD in individual infants. (b) Histogram of the optimal TE calculated for each voxel (range: 30–70 ms). The average optimal TE = 52 ms. BOLD, blood oxygen level dependent; EPI, echo planar imaging; TE, echo time.

A voxel‐wise regression analysis was also performed across the whole brain. A second‐order polynomial was fitted to the data within each voxel using a general linear model. The model fit was then used to find the TE at which the maximal percentage change in BOLD signal was detected in each voxel. The optimal TE across all voxels of the brain was used to generate a histogram and report the average optimal TE (see Fig. 1B). The 25th and 75th percentiles were reported as a range for the optimal TE.

In addition to the whole brain analysis, four ROIs were selected in order to investigate the regional variation in TE across different brain areas. The ROIs chosen are known to be strongly activated by noxious stimuli 30, 31. Anatomical ROIs were classified based on a neonatal‐specific atlas 32 and projected to each subject's individual functional space, as described for the whole brain mask. The selected ROIs were the bilateral anterior cingulate cortex (ACC), insula, postcentral gyrus, and thalamus. Once transformed, all masks were thresholded for partial volume, as described for the whole brain mask above. The eroded and thresholded whole‐brain mask (in functional space) was used to select overlapping voxels within each ROI mask, ensuring that voxels falling outside of the brain were removed.

Group analyses were conducted across the whole brain using a fixed effects analysis in FEAT (see Supporting Fig. S1). Data were grouped according to the TE used during acquisition. Due to the different numbers of data points at each TE, group images depicting the percentage contrast of parameter estimates (COPE) are shown. A map of the percentage COPE at each TE was calculated by using fslmaths to divide the group COPE image (generated by the fixed effects group analysis) by a scaling factor derived using Featquery.

#### Study 2: Quantitative $T_{2}^{*}$ Relaxometry

From the seven infant's data included in Study 2, 84 volumes of EPI data in total were collected. Each subject's individual EPIs were motion‐corrected with linear registration using MCFLIRT 33. Following this, five out of 84 volumes were excluded from further analysis due to excessive motion or distortion artifacts.

The observed signal from a gradient echo EPI BOLD experiment as a function of TE can be described by the monoexponential decay equation (Eq. [1]):

(1) $$S=S_{0}e^{\left(-\frac{TE}{T_{2}^{*}}\right)},$$

where *S* is the measured MR signal and *S*_*0*_ represents the underlying MR signal without $T_{2}^{*}$ weighting, which is proportional to proton density, receive coil array sensitivity, and RF amplifier scaling factors. Using a voxel‐wise regression analysis across the signals from all measured TEs, the above equation was used to obtain an estimate of the $T_{2}^{*}$ for each voxel (regression conducted in MatLab [Mathworks, Natick, MA]). This $T_{2}^{*}$ map of the brain was subsequently used to calculate mean $T_{2}^{*}$ values for the whole brain and the four ROIs (ACC, insula, postcentral gyrus, and thalamus). The same registration methods, templates, and anatomical masks were used as described for Study 1. Regions of interests from the infant atlas were similarly transformed to EPI space using the transforms generated from individual registrations, and average $T_{2}^{*}$ values were calculated in each ROI. Prior to computing the average, outlier values were removed using the following equations: outliers ≤ Q1 − 1.5*(Q3 − Q1) and outliers ≥ Q3 + 1.5*(Q3 − Q1), where Q1 and Q3 refer to the first and third quartiles calculated from the data and (Q3 − Q1) denotes the interquartile range. Finally, $T_{2}^{*}$ values were averaged across participants to produce means for each ROI.

## RESULTS

The mean percentage change in BOLD activity across the whole brain at five TEs between 30 and 70 ms is shown in Figure 1A (Study 1). Based on the second‐order polynomial regression, the maximal sensitivity to BOLD signal occurred at a TE of 52 ms. Group analyses investigating the changes in BOLD activity across the whole brain at different TEs are presented in Supporting Figure S1. A TE of 50 ms provided the greatest sensitivity to changes in BOLD in this population.

In addition, a voxel‐wise regression analysis was carried out across the whole brain to identify the optimal TE in each individual voxel, which identified that 52 ms was the average optimal TE (see Fig. 1B). The interquartile range from the voxel‐wise analysis was 48 to 55 ms, suggesting a suitable range for the optimal TE. Furthermore, the relationship between the CNR and TE was also considered across the whole brain. Consistent with the previous observations, an optimal TE of approximately 50 ms was identified.

**Figure 2:**
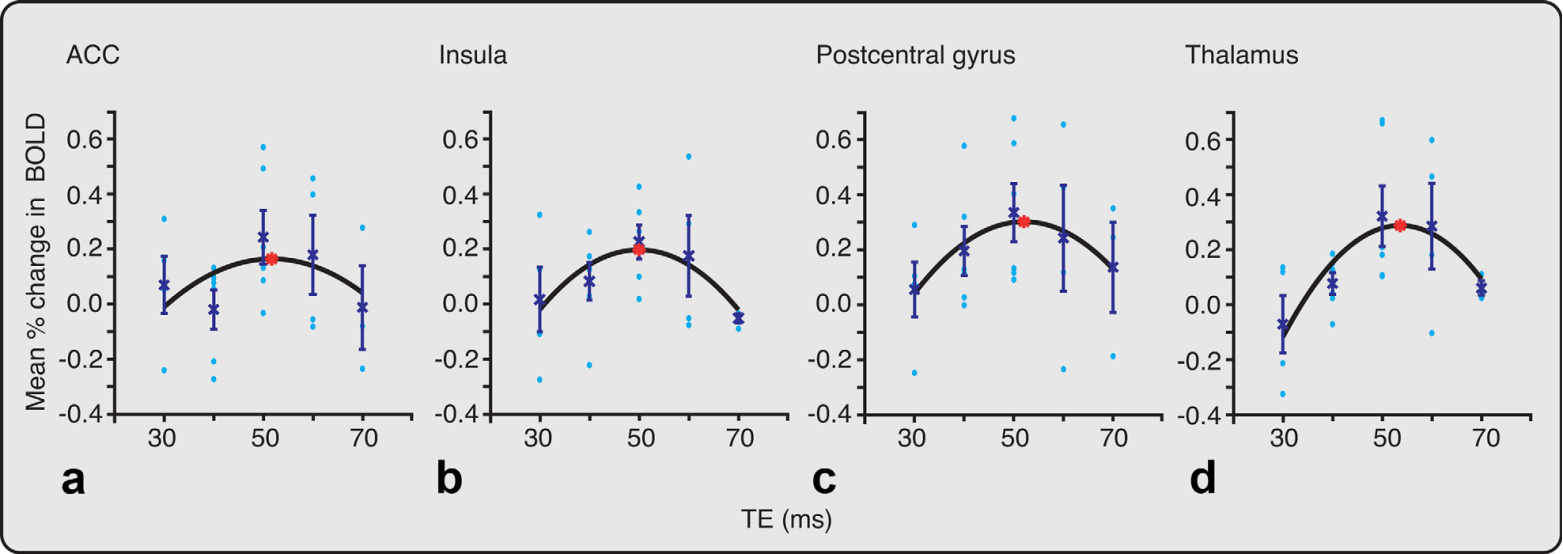
Optimal TE investigated within four ROIs. The mean percentage change in BOLD at different TEs are reported in the bilateral (a) ACC, (b) insula, (c) postcentral gyrus, and (d) thalamus. The mean (crosses) and standard error of the mean are indicated at each TE. A second‐order polynomial was fitted to the data (black line). In each ROI, an asterisk (red) identifies the TE at which the fitted curve is maximal. The TE that generates the maximal mean percentage changes in BOLD signal is given in Table 2. The circles (light blue) represent the mean percentage change in BOLD in individual infants. ACC, anterior cingulate cortex; BOLD, blood oxygen level dependent; EPI, echo planar imaging; ROI, region of interest; TE, echo time.

The optimal TE was also investigated in four different ROIs: the anterior cingulate cortex (ACC), insula, postcentral gyrus, and thalamus. Figure 2 shows the mean percentage change in BOLD at TEs between 30 and 70 ms for each ROI. In each of the four ROIs, the maximal percentage change in BOLD occurred between 50 and 54 ms (see Fig. 2 and Table 2). The greatest percentage change in BOLD was observed in the thalamus and postcentral gyrus, suggesting that noxious stimulation in infants evoked particularly strong activation in these brain regions.

**Table 2:** Optimal TE Within the Whole Brain and Four Regions of Interest

Region of Interest	TE (ms)	Mean % Change in BOLD
Whole brain	52.0	0.19
Anterior cingulate cortex	51.7	0.16
Insula	50.0	0.20
Postcentral gyrus	52.1	0.30
Thalamus	53.6	0.29

The TE at which the maximal mean percentage change in BOLD signal was observed is reported across the whole brain and in four ROIs (see also Figs. 1 and 2).

BOLD, blood oxygen level dependent; ROI, region of interest; TE, echo time.

It was also of interest to compare the $T_{2}^{*}$ values to the optimal TE calculated within the same brain regions in infants. In Study 2, average $T_{2}^{*}$ was calculated from the signal collected at six TEs (TE range: 30–80 ms). $T_{2}^{*}$ values within the whole brain and the four ROIs are reported in Table 3. Although the TEs reported in Study 1 are similar across all brain regions, there was more variation in the $T_{2}^{*}$. The average $T_{2}^{*}$ in the deeper brain regions (ACC, insula, and thalamus) was longer compared with the more superficial postcentral gyrus. Overall, $T_{2}^{*}$ values are approximately double those reported in adults 4, 5, 6.

**Table 3:** Average $T_{2}^{*}$ Within the Whole Brain and Four Regions of Interest

Region of Interest	Mean $T_{2}^{*}$ ± SD	Range of Mean Values Across Participants
Whole brain	99 ± 8	91–115
Anterior cingulate cortex	113 ± 18	96–150
Insula	131 ± 11	116–149
Postcentral gyrus	87 ± 6	78–96
Thalamus	106 ± 8	98–122

Mean $T_{2}^{*}$ values calculated across the whole brain and in four different ROIs from infant $T_{2}^{*}$ maps (n = 7).

ROI, region of interest; SD, standard deviation.

Review article carefully. As needed, abbreviations deleted at only mention and expanded at first mention.

## DISCUSSION

The aim of this study was to identify the TE that allows the maximum change in BOLD signal to be recorded following the application of an experimental noxious stimulus in term‐born infants. Because the optimal TE is theoretically equivalent to $T_{2}^{*}$ 1, 2, 3, for comparison the $T_{2}^{*}$ values were also calculated. Here we provide evidence that a TE of approximately 50 ms should be used in infant fMRI experiments conducted at a field strength of 3T.

Following the application of a controlled noxious experimental stimulus across a range of TEs from 30 to 70 ms, the maximal change in BOLD signal was recorded at a TE of approximately 50 ms. This TE is approximately 20 ms longer than the TE commonly used in adult studies, which demonstrates the importance of not assuming that imaging parameters optimized in the adult population are appropriate for use in investigations in infants. Direct extrapolation of the adult TE would result in a marked reduction in the magnitude of the BOLD signal recorded in infants. However, although this study indicates that optimal TE is age‐dependent and longer in term infants compared with adults, it is not known how the optimal TE varies during childhood or in infants who have been born prematurely. Moreover, the voxel‐wise analysis presented here suggests that a TE of exactly 50 ms in term infants is not critical, and some studies may favor slightly shorter or longer TEs.

The average $T_{2}^{*}$ value across the whole infant brain was approximately 100 ms, which is similar to the $T_{2}^{*}$ value recently reported at 3T in the motor cortex in a single infant 12. This is substantially longer than $T_{2}^{*}$ values reported in adults, where values from 41 to 66 ms have been observed in gray and white matter regions 4, 5, 6. Our results are consistent with previous studies in adults in which the $T_{2}^{*}$ is approximately double the TE used in practice. The discrepancy between the $T_{2}^{*}$ values and the optimal TE is thought to arise due to intrinsic thermal noise in the scanner setup and physiological noise, such as changes in heart rate and breathing rate 4. In addition, it is important to note that $T_{2}^{*}$ measurements are dependent on voxel size; therefore, the use of smaller voxels may favor an even longer optimal TE.

During the postnatal period, the infant brain undergoes considerable growth and development. Changes in synaptogenesis, synaptic pruning, and myelination of white matter tracts contribute to an increase in proton density and concentration of macromolecules within the brain tissues 10, 34. The infant brain also has higher water content and lower lipid concentrations compared with adults 9, 35, which are largely driven by the limited myelin present. Furthermore, histology and MRI studies show low iron concentration in the infant brain, which is found in much higher concentrations in the adult brain 36, 37. The differences in brain structure and composition are thought to contribute to the observed differences in the optimal TE and $T_{2}^{*}$ between the infant and adult populations 11.

In adults, regional variation in $T_{2}^{*}$ has been reported. For example, $T_{2}^{*}$ values of 66 ms in cortical gray matter, 53.2 ms in cortical white, and 31.5 ms in the putamen have been observed 6. Similarly, variation in the optimal TE across different brain regions has also been reported. An optimal TE between 25 and 30 ms has been observed in the visual cortex compared with a TE between 30 and 45 ms in the auditory and motor cortices 29. In infants, small regional variations in $T_{2}^{*}$ values were also observed, with longer $T_{2}^{*}$ values in deeper brain regions compared to the more superficial postcentral gyrus. However, we did not observe this degree of regional variation in optimal TE across different brain regions. It is likely that the composition of the tissue in the infant brain is more homogenous compared with the adult. Although the number of brain regions investigated here was small, the relatively uniform optimal TE observed across the whole brain suggests that a TE of approximately 50 ms would be appropriate when considering other specific ROIs.

In this study, the optimal TE was identified in response to repeated experimental noxious stimuli. At a similar interstimulus interval, electrophysiological recordings in term infants do not show habituation of the evoked brain activity 38. It is therefore unlikely that habituation of the signal substantially influenced the maximum change in BOLD response. It is not possible to conclude whether the TE reported here would be optimal in experiments using alternative stimulus modalities and during resting state. However, in practice a single TE is commonly used during adult fMRI experiments across a range of stimulus modalities 7 and in resting state investigations 8. The effect of physiological and thermal noise and scanning acquisition parameters, such as acquisition bandwidth, on the BOLD signal is unlikely to be dependent on stimulus modality; and an advantage of using a noxious stimulus is that multiple areas of the brain are activated. The use of these optimized scanning parameters will be particularly useful in studies investigating infant pain. In preverbal infants, the measurement of pain and the provision of appropriate analgesic treatment is a challenge 22. Functional MRI may provide a useful surrogate measure of analgesic efficacy in this population 20, 21, 22.

## CONCLUSION

In summary, this study systematically investigated the optimal TE for measuring BOLD responses in infants at 3T. The optimal TE was found to be approximately 50 ms, which we recommend for use in future 3T fMRI investigations in term‐born infants.

## Supporting information

Additional supporting information may be found in the online version of this article.

**Fig. S1.** Maps of the percentage change in the contrasts of parameter estimates (COPE) at different TEs. (A) TE = 30 ms (n = 4); (B) 40 ms (n = 6); (C) 50 ms (n = 6); (D) 60 ms (n = 4) and (E) 70 ms (n = 3). A red‐yellow colour scale represents positive parameter estimates (thresholded at percentage COPE > 0.2). MNI coordinates (top) describe the locations of each plane in mm.
