# Supplementary material for: Optimal echo time for functional MRI of the infant brain identified in response to noxious stimulation
Source: Magn Reson Med. 2016 Sep 21;78(2):625–31. doi: 10.1002/mrm.26455 (PMC5516146; doi:10.1002/mrm.26455)
Supplement: Supplementary file 1 — Fig. S1. Maps of the percentage change in the contrasts of parameter estimates (COPE) at different TEs. (A) TE = 30 ms (n = 4); (B) 40 ms (n = 6); (C) 50 ms (n = 6); (D) 60 ms (n = 4) and (E) 70 ms (n = 3). A red‐yellow colour scale represents positive parameter estimates (thresholded at percentage COPE > 0.2). MNI coordinates (top) describe the locations of each plane in mm. [file MRM-78-625-s001.docx]

**Supporting Material**

Supporting Figure S1: Maps of the percentage change in the contrasts of parameter estimates (COPE) at different TEs. (A) TE = 30 ms (n = 4); (B) 40 ms (n = 6); (C) 50 ms (n = 6); (D) 60 ms (n = 4) and (E) 70 ms (n = 3). A red-yellow colour scale represents positive parameter estimates (thresholded at percentage COPE > 0.2). MNI coordinates (top) describe the locations of each plane in mm.
